# Evaluation Survey on Agreement with Existing Definitions of Biosecurity with a Focus on Livestock

**DOI:** 10.3390/ani13091518

**Published:** 2023-04-30

**Authors:** Claude Saegerman, Gianni Parisi, Jarkko Niemi, Marie-France Humblet, Jorge Ron-Román, Bachir Souley Kouato, Alberto Allepuz, Vincent Porphyre, Maria Rodrigues da Costa, Véronique Renault

**Affiliations:** 1 Research Unit in Epidemiology and Risk Analysis Applied to Veterinary Sciences (UREAR-ULiege), Fundamental and Applied Research for Animal Health (FARAH) Centre, Faculty of Veterinary Medicine, University of Liege, 4000 Liege, Belgium; 2 Unit of Faculty Biosecurity, Faculty of Veterinary Medicine, Liege University, 4000 Liege, Belgium; 3 Bioeconomy and Environment Unit, Natural Resources Institute Finland (Luke), 60320 Seinäjoki, Finland; 4 Biosafety and Biosecurity Unit, Department of Occupational Safety and Health, University of Liege, 4000 Liege, Belgium; 5 Grupo de Investigación en Sanidad Animal y Humana (GISAH), Carrera de Ingeniería en Agropecuaria, Departamento de Ciencias de la Vida y la Agricultura, Universidad de las Fuerzas Armadas-ESPE, Sangolquí P.O. Box 171-5-231, Ecuador; 6 Institut National de la Recherche Agronomique du Niger (INRAN), Niamey P.O. Box 429, Niger; 7 Department of Animal Health and Anatomy, Universitat Autònoma de Barcelona (UAB), 08193 Barcelona, Spain; 8 CIRAD, UMR SELMET, F-34398 Montpellier, France; 9 CIRAD, INRAE, Institut Agro Montpellier, Université de Montpellier, SELMET, F-34398 Montpellier, France; 10 Centre for Epidemiology and Planetary Health (CEPH), Scotland’s Rural College (SRUC), Inverness Campus, Inverness IV2 5NA, UK; 11 Agronomes et Vétérinaires Sans Frontières, 69007 Lyon, France

**Keywords:** survey, biosecurity, livestock, definition, stakeholder, agreement, One Health, EU COST Action

## Abstract

**Simple Summary:**

Disease prevention, including biosecurity, surveillance, and traceability are key aspects to minimize the risk of animal diseases causing harm to society. Diseases for which biosecurity are needed depend on species of interest, e.g., African swine fever, avian influenza, or foot-and-mouth disease. However, several definitions of biosecurity co-exist in the literature. A survey was set up to investigate the level of agreement of participants regarding eight existing definitions of the (livestock) biosecurity, to rank keywords to consider before attempting a more consolidated definition, and to select the desirable qualities of a definition of livestock biosecurity. Respondents had a male–female gender ratio close to one, were mostly between 25 and 54 years old, and had animal health as the main first field of expertise (30% were government officials). The significant most popular biosecurity definition was the one that conceptualized the rules of 5B’s (bio-exclusion, bio-containment, bio-compartmentation, bio-prevention, and bio-preservation). The top two keywords to consider for the consolidation of the biosecurity definition were “prevention” and “measures”. The optimal biosecurity definition needs to be operational and related to animal health but also comprehensible, simple, and related to public health. The survey results highlight the need for the integration of more aspects in the existing definitions of livestock biosecurity (e.g., prevention of zoonoses and preservation of the environment and diversity).

**Abstract:**

Disease prevention, including biosecurity, surveillance, and traceability are key aspects to minimize the risk of animal diseases causing harm to society. Diseases for which biosecurity are needed depend on species of interest, e.g., African swine fever, avian influenza, or foot-and-mouth disease. However, several definitions of biosecurity co-exist in the literature. Under the new COST Action “Biosecurity Enhanced Through Training Evaluation and Raising Awareness” (BETTER) CA20103, we launched an initial survey on the agreement with eight existing definitions of (livestock) biosecurity, to rank keywords to consider before attempting a more consolidated definition, and to select the desirable qualities of a definition of livestock biosecurity. Respondents (N = 316) had a male–female gender ratio close to one, were mostly between 25 and 54 years old, and had animal health as the main field of expertise (30% were government officials). The significant most popular biosecurity definition was the one that conceptualized the rules of 5B’s (bio-exclusion, bio-containment, bio-compartmentation, bio-prevention, and bio-preservation). The top two keywords to consider for the consolidation of the biosecurity definition were “prevention” and “measures”. The optimal biosecurity definition needs to be operational and related to animal health but also comprehensible, simple, and related to public health. The survey results highlight the need for the integration of more aspects in the existing definitions of livestock biosecurity (prevention of zoonoses and preservation of the environment and diversity).

## 1. Introduction

**Figure 1:**
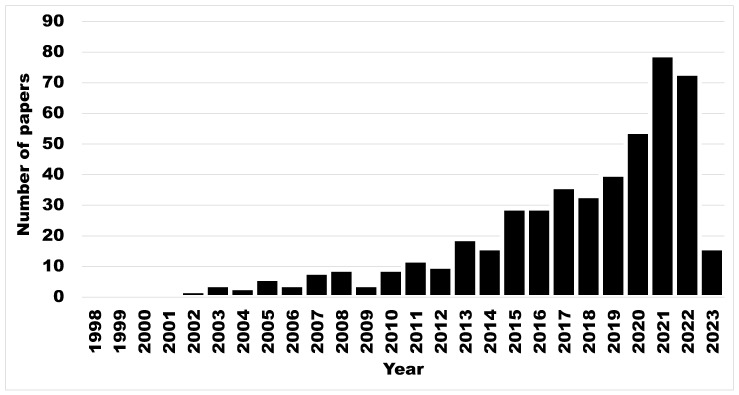
Number of papers (Y-axis) present in PubMed (US National Library of Medicine, National Institutes of Health) mentioning “livestock” and “biosecurity”, in function of time (X-axis), 1998–2023 (N = 433). Legend: 2023, situation at 10 April.

The European Animal Health Law (Regulation (EU) 2016/429) emphasized disease prevention, including biosecurity, surveillance, and traceability, as key aspects to minimize the risk of animal diseases causing harm to society [1]. Livestock biosecurity gained increasing attention during the last decades. Diseases for which biosecurity are needed depend on species of interest, e.g., African swine fever, porcine epidemic diarrhea, avian influenza, or foot-and-mouth disease [2,3,4]. The results of a search string conducted in PubMed (US National Library of Medicine, National Institutes of Health) using the following search keys and Boolean operator on 10 April 2023 ((biosecurity [Title/Abstract]) AND (livestock [Title/Abstract])) showed that an annual increasing number of articles on biosecurity (N = 433) were published during 1998–2023, including 83 review papers, but no meta-analyses (Figure 1).

A recent short communication reviewed the origins and evolution of the biosecurity concept and discussed the future perspectives of biosecurity concerning the One Health approach and the changing environment [5]. The implementation of this broader concept of biosecurity will need a strengthened collaboration and interaction among the different sectors at all levels, which represents a major challenge [5]. Intersectoral collaboration is related to the engagement of stakeholders, including farmers and private veterinarians in livestock biosecurity. A stakeholder is defined as an “individual, group of persons or organization that can affect or is affected by the decisions of another organization, including interest groups related to the organization. A stakeholder’s relationship with the focal organization is generally determined by three main attributes, i.e., the power to influence the organization, a legitimate relationship with the organization, and an urgent claim on the organization” [6]. Engaging stakeholders, including farmers and private veterinarians concerned and involved or interested by livestock biosecurity is fundamental to improve the quality of biosecurity, to strengthen public trust in governance and to enhance compliance (observance) with biosecurity measures [7]. In addition, a broad participation (and support) is expected to include opinions in their diversity at an international level, considering the issues to be faced not only in Europe, but also in developing and transitioning countries [8].

To initiate engagement of stakeholders, a first step is to obtain a consensus about what is livestock biosecurity. In fact, when a consensus on the definition of biosecurity with a focus on livestock biosecurity is obtained, it will be easier for all to understand the objectives to reach, to engage stakeholders in the same direction, to enhance compliance of biosecurity, and to foster communication about biosecurity. In the past, the definition of biosecurity was almost exclusively related to internal and the external biosecurity (e.g., [9]) and less to broader aspects of biosecurity, such as the prevention of humans against zoonoses, or the impact of biocide use on the environment [5]. In order to capture other new dimensions to integrate in the biosecurity concept, it is also important to consider not only the definition of livestock biosecurity, but to open the door for wider definitions related to biosecurity in general. Recent opinion/review papers suggest a more unified concept of biosecurity to integrate human, animal, plant, and environmental health [10,11].

Several definitions of biosecurity coexist in the literature. Under the new COST Action “Biosecurity Enhanced Through Training Evaluation and Raising Awareness” (BETTER) CA20103 (https://better-biosecurity.eu/; accessed on 15 April 2023) we launched an initial survey on the participant’s agreement with definitions of biosecurity (i.e., involved or interested in livestock biosecurity).

The aim of this survey was to improve the knowledge of (i) the level of agreement of participants regarding eight existing definitions of biosecurity with a focus on livestock biosecurity; (ii) to rank keywords to consider before attempting a more consolidated definition of livestock biosecurity; and (iii) to select the desirable qualities of a definition of livestock biosecurity.

## 2. Materials and Methods

### 2.1. Study Design and Sampling

An online cross-sectional survey was set up to (i) investigate the level of agreement of participants regarding eight existing definitions of (livestock) biosecurity that were extracted from various sources and publications using a recent review [5] (Table 1); (ii) to rank keywords to consider before attempting a more consolidated definition of livestock biosecurity; and finally, (iii) to select desirable qualities of a definition of livestock biosecurity. The questions were developed by taking into account results of the first brainstorm between the four first and the last author. The existing definitions of biosecurity were identified based on a literature search.

The survey was distributed to diverse persons interested or involved in biosecurity, especially livestock biosecurity, i.e., contact points by continent and in different existing networks were contacted, such as the EU COST Action BETTER dedicated to livestock biosecurity; the already completed EU COST Action Cystinet dedicated to Taeniosis/Cysticercosis; and the EU COST Action ASF-STOP dedicated to African swine fever; the Emerging Risks Exchange Network of European Food Safety Authority; the European Veterinary Association; the European Federation for Animal Health and Sanitary Security; and the European Association of Establishments for Veterinary Education; the National Institute for Animal Agriculture in United States of America; and institutions/non-government organizations involved in developing countries (Cirad, VSF, and VASF). The same persons were asked to circulate the questionnaire to their networks to reach the persons interested or/and involved in (livestock) biosecurity using a snowball sampling strategy [12]. This strategy was used, as no sampling frame of those persons was available.

**Table 1:** Eight definitions of the biosecurity considered in this survey.

Code	Definition
A	A strategic and integrated concept that encompasses the policy and regulatory frameworks (including instruments and activities) that analyse and manage risk in food safety, public health, animal life and health, and plant life and health, including associated environmental risk [13].
B	The sum of management and physical measures designed to reduce the risk of the introduction, development, and spread of diseases to, from, and within: (a) an animal population, or (b) an establishment, zone, compartment, means of transport or any other facilities, premises, or location [1].
C	The prevention of misuse through loss, theft, diversion, or intentional release of pathogens, toxins, and any other biological materials [14].
D	The vital work of strategy, efforts, and planning to protect human, animal, and environmental health against biological threats [15].
E	The strategies to assess and manage the risk of infectious diseases, quarantine pests, invasive alien species, living modified organisms, and biological weapons [16].
F	A unified concept to integrate human, animal, plant, and environmental health [10].
G	All measures to prevent the introduction of pathogens (bio-exclusion) and reduce the spread of pathogens (bio-containment) [17].
H	All measures: (1) to limit the risk of introduction (bio-exclusion); (2) to limit the spread of the pathogen within the same facility, e.g., by isolating excreting animals (bio-compartmentation); (3) to limit the spread of the disease agent outside the facility (inter-herd transmission) (bio-containment); (4) to prevent the risk of human contamination (bio-prevention); and (5) to prevent any environmental bio-contamination and persistence of the pathogen (bio-preservation) [18].

### 2.2. Data Collection and Survey

The responses were collected in an anonymous online survey that was created, hosted, and shared using the LimeSurvey^®^ software (version 2.06+). The survey questionnaire (Appendix A) was divided into four sections, each with a subset of questions: (i) socio-demographic characteristics of the respondents (eight questions); (ii) score of agreement with different definitions using a scale from 0 (fully disagree) to 10 (fully agree) (the order of appearance of definitions was at random to avoid bias); (iii) important keywords to consider for a further consolidated definition of livestock biosecurity (at least one and at maximum three keywords in decreasing order); and (iv) the desirable quality of an optimal definition of livestock biosecurity on the side of respondent.

Concerning the socio-demographic characteristics of the respondents, the age of the respondents, as well as their country of origin, their jobs could determine the way they experience biosecurity. They face different realities and that could therefore have an influence on the way the concept of biosecurity is perceived. This information was collected to detect such potential disparities and to see if such disparities could lead to bias due to the overrepresentation of some groups.

The questionnaire was launched on 20 May 2022, and was open to responses until 22 June 2022. It was anonymous, did not include personal or sensitive data, and according to the European legislation, did not specifically require approval by an Ethical Committee. However, the data protection officer of the University of Liège validated the questionnaire before its distribution to the potential respondents.

### 2.3. Definition of Biosecurity

Eight definitions were extracted from various sources and publications using a recent review [5] (Table 1).

### 2.4. Data Analysis

Responses were extracted from the LimeSurvey^®^ (version 2.06+) application to a Microsoft Excel^®^ spreadsheet for analysis. Only complete questionnaires were processed for analysis. Data were cleaned and records were deleted if the respondent did not complete the questionnaire.

The score of agreement with biosecurity definitions was estimated by the participants using a scale from 0 (fully disagree) to 10 (fully agree). Violin plots were used to represent the level of agreement by definition. Violin plots are similar to box plots (vertical axis), except that they also show the probability density of the data at different values (horizontal axis), usually smoothed by a kernel density estimator. To estimate if significant differences existed between the level of agreement in regard to definitions, a quantile regression was applied. A two-sample Wilcoxon rank sum (Mann–Whitney) test was used to compare the level of agreement for the most popular definition between European and non-European countries (as Europe is the most represented continent in the sample), between Belgium and other European countries (as Belgium is the European country most represented in the sample), and between participants involved or not specifically in biosecurity [19].

Open-ended questions were sorted manually and summarized in an interpretative way. All statistical analyses were conducted using Microsoft Excel^®^ and STATA S.E. 14.2^®^ software (College Station, TX, USA). The limit of significance for all tests was 0.05.

## 3. Results

### 3.1. Survey Response

**Figure 2:**
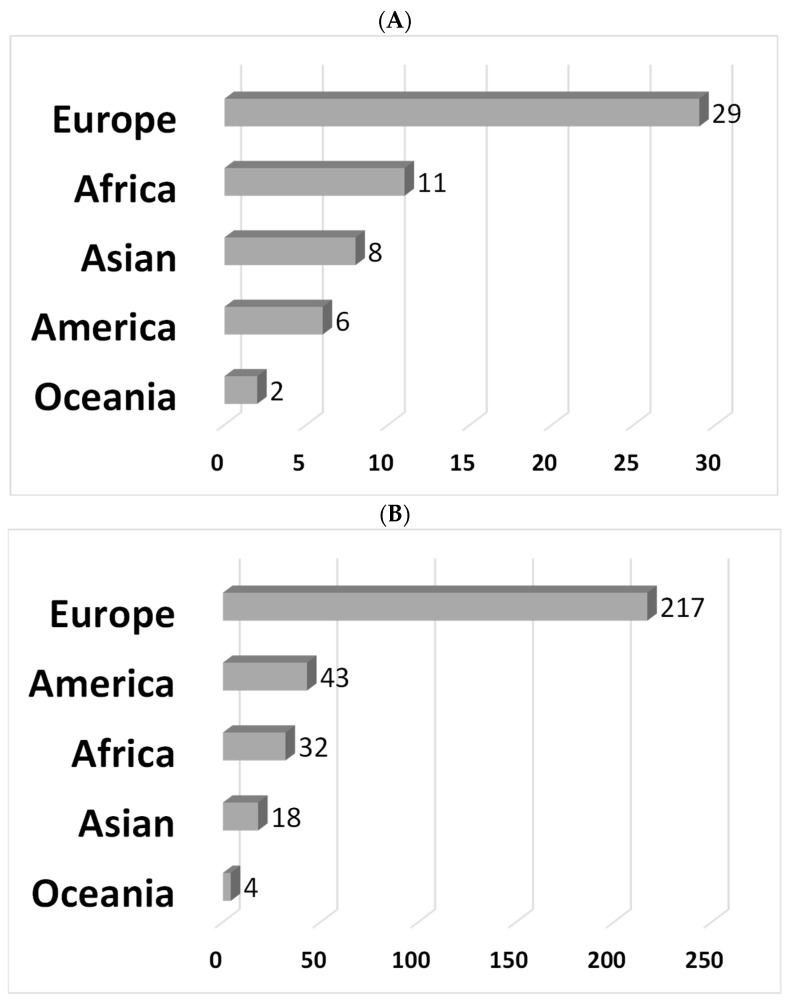
Number of countries by continent (N = 56) (**A**) and number of respondents by continent (N = 314), and (**B**) by continent. Legend: Two respondents were not assigned to any country due to the international character of their activities (FAO). Details of respondents by country are depicted in Appendix B.

The questionnaire was opened by 527 people. We assumed that this number corresponded to the number of people reached in one month—the period of time the survey was open (i.e., people interested or involved in matters concerning biosecurity). After cleaning and deleting incomplete records, a total of 316 respondents completed the survey (i.e., 60%, 316/527), coming from 56 countries and 5 continents (Figure 2). The most represented country in the sample was Belgium (14.9% of the sample, 21.7% of European responses).

### 3.2. Socio-Demographic Characteristics of Respondents

The socio-demographic characteristics of respondents are depicted in Table 2. Respondents were characterized by a male–female gender ratio close to one, were mostly between 25 and 54 years old, and had animal health as their main field of expertise. Half of respondents (N = 160) were specifically involved in biosecurity, mostly categorized as working for government officials (30%) or workers (26%). Of all 160 stakeholders involved in biosecurity, 42% and 58% were involved or not in COST-Action BETTER.

### 3.3. Level of Agreement with Existing Livestock Biosecurity Definitions

**Figure 3:**
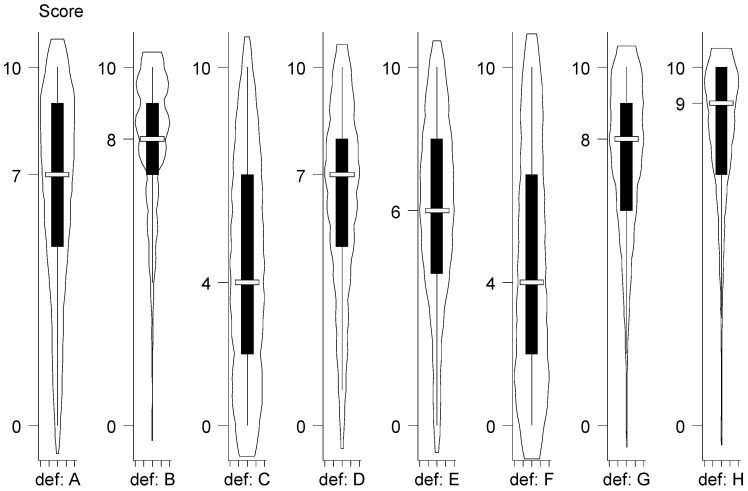
Violin plot of the score of agreement with eight existing definitions of livestock biosecurity (N = 316). Legend: Violin plots are similar to box plots (vertical axis), except that they also show the probability density of the data at different values (horizontal axis), usually smoothed by a kernel density estimator. The violin plot displays the median as a short horizontal line, the first-to-third interquartile range as a narrow-shaded box, and the lower-to-upper adjacent value range as a vertical line, but it does not plot outside values. The score of agreement was estimated by participants using a scale from 0 (fully disagree) to 10 (fully agree); def. = definitions A, B, C, D, E, F, G, and H refer to the definition in Table 1.

The agreement of the participants with eight existing definitions of biosecurity is presented in the Figure 3.

With definition A as reference and using quantile regression, we found significant lower agreement for the definitions C, D, E, and F (*p*-value ≤ 0.001), but significant higher agreement for the definitions B, G, and H (*p*-value ≤ 0.001). The definitions B, G, and H can therefore be considered as the most popular with the definition H having an agreement score significantly higher than the other two (*p*-value < 0.001). For definition H, no effect of origin was demonstrated, i.e., European versus a non-European country (two-sample Wilcoxon rank sum test; *p*-value = 0.69), and Belgium versus other European countries (two-sample Wilcoxon rank sum test; *p*-value = 0.53). In addition, no effect was demonstrated for definition H if we tested the group of people involved in biosecurity versus other participants interested in biosecurity (two-sample Wilcoxon rank sum test; *p*-value = 0.18), the group of respondent active in animal health versus other activities (two-sample Wilcoxon rank sum test; *p*-value = 0.29), the group of members of scientific (institution) and/or academic (university/school) staff versus other category of respondents (two-sample Wilcoxon2 rank sum test; *p*-value = 0.30), the four age groups (18 to 24 years; 25 to 39 years; 40 to 54 years; and 55 years and over) of respondents (Kruskal–Wallis equality of populations rank test; *p*-value = 0.29), or if we tested the group of government officials versus other categories of stakeholders involved in the survey (two-sample Wilcoxon rank sum test; *p*-value = 0.54).

### 3.4. Keywords to Consider for the Consolidation of the Definition of Livestock Biosecurity

Respondents gave at least one keyword and alternatively a maximum of two other keywords that need consideration for a further consolidation of the definition of livestock biosecurity (Table 3). Keywords that were cited at least 10-fold, as first, second, or third position are depicted in Table 3. The top two keywords to consider for the consolidation of the definition of livestock biosecurity were in decreasing order: prevention (*n* = 155 occurrences; Poisson regression, *p*-value < 0.001) and measures (*n* = 24 occurrences; Poisson regression, *p*-value = 0.02). The most counted keyword “prevention” was not affected by the country of origin of the respondent (Firthlogit regression; *p*-value > 0.229). Testing the influence of a country of origin on other keywords proposed was not possible due to lack of power.

### 3.5. Desirable Qualities of Biosecurity Definitions

The characteristics of an optimal definition of livestock biosecurity are depicted in Table 4. Participants highlighted the most important characteristics for an optimal definition of livestock biosecurity that should be operational (72.8% of respondents) and related to animal health (64.2% of respondents). Around 50% of respondents also considered that it should be comprehensible, simple, and related to public health.

## 4. Discussion

Developing a consensus to the definition of livestock biosecurity is challenging and it is one of the tasks that the BETTER COST Action (https://better-biosecurity.eu/; accessed on 15 April 2023) is conducting. To initiate this process, we designed and implemented an initial cross-sectional survey with eight existing definitions of biosecurity. Several methods to obtain consensus on definitions exist, such as Delphi, Nominal Group, and models developed by the National Institutes of Health and Glaser (e.g., [11,20]). Each method needs time and has advantages and disadvantages in comparison to others (for a review, see [20,21]).

In this initial cross-sectional survey, we captured the preference of over three hundred people involved or interested in (livestock) biosecurity worldwide. We opted for the use of a score of agreement with biosecurity definitions using a scale from 0 (fully disagree) to 10 (fully agree). In the online survey, the visualization of the scoring system allows the comparison between the score for each definition. This methodology allows each respondent to have a relative cross-check between all definitions. To aggregate the score of all respondents, a violin plot representation was used because it allows for visualization of the distributions of numeric data (score; vertical axis) for the different definitions using density curves (horizontal axis). In addition, to capture any significant differences in the agreement scores of the definitions, a quantile regression was applied.

This survey provides relevant indication in terms of preference from existing definitions of biosecurity. Among eight existing definitions of biosecurity, three were markedly better scored, composing the top three. These three most popular definitions have common elements when compared to the other definitions presented in the survey. They have clarity and are operational, as suggested by the desirable qualities of a definition (see after). They are also more specific (i.e., emphasize animals/animal production more) than the definitions that were less popular.

Among these three definitions, the definition H [18] obtained agreement scores significantly higher compared to definitions B and G [1,17], and there was no difference between responses from European and non-European countries, or between Belgium and other European countries, indicating that the overrepresentation of Europe and Belgium does not seem to affect the conclusion. No difference was found either between stakeholders involved in and interested in biosecurity, between respondents coming from institutions/universities and other origins, or between government officials and other categories of stakeholders involved in the survey. This definition presents the conceptualization of the rule of 5 Bs (bio-exclusion, bio-containment, bio-compartmentation, bio-prevention, and bio-preservation). This definition is broader and includes clearly the prevention of zoonoses by the operator and the bio-preservation to avoid bio-contamination and persistence of pathogens in the environment [22,23]. Regarding the prevention of zoonotic diseases, a systematic review highlighted the need for biosecurity measures (hygienic measures, use of personal protective equipment) (e.g., [22]). Several factors are of importance for biosecurity measures against zoonoses to be applied. Some of these factors, such as the risk susceptibility and the benefits of the measures, could be influenced by evidence-based communication [23]. In addition, preservation of the environment was also highlighted in livestock biosecurity [24].

Both the second (B) and the third (G) best-scored definitions of biosecurity have the same median preference from respondents. The second is the definition of biosecurity in the Animal Health Law [1]. The third is the definition of the OIE-FAO [17,25]. Both are restricted to limit the introduction and the spread of pathogens, but the second definition is more precise on the scale of biosecurity (animal population, establishment, zone, compartment, means of transport, or any other facilities, premises, or location).

In the future, the importance of biosecurity in mitigating the risks for animal and public health and environmental contamination will have to be further developed and taken into account [5]. It can support the One Health biosecurity concept.

Regarding keywords to be considered for further consolidation of the definition of livestock biosecurity, the translation of biosecurity in legislation received little support (i.e., 27.8% of respondents). This result might be related to the debate among the feasibility of establishing by law mandatory biosecurity measures. As a matter of fact, according to FESASS, the approach of using a methodology (e.g., www.mijnmaniervanwerken.be) rather than imposing strict or detailed rules is preferable [26]. This explains why in the legislative framework, it is very difficult to reach a consensus on the minimum level of biosecurity to gain [27]. Among the preferred key words by respondents, the cornerstone was the “prevention”, and secondly, “measures”. Prevention is better than cure and contributes to the global and national security [28]. This cornerstone is thus expected and needs full consideration to consolidate the definition of livestock biosecurity. In addition, and due to the fact that the effect of the country of origin was tested only for the most counted keyword (“prevention”), no definitive inference can be made about a “more consolidated” definition of biosecurity. More surveys are necessary to debate this topic in all continents with a large number of stakeholders.

On top of the desirable characteristics, participants considered that the optimal definition of livestock biosecurity should be operational and related to animal health. Indeed, all three top three ranked existing definitions could be considered as such, and they also included the two top keywords (i.e., prevention and measures). The most popular existing definition (i.e., definition H) might be considered an operational definition. For example, it is used in standard operating procedures (SOPs) in all clinics in the Faculty of veterinary medicine of the Liège University (https://www.fmv-biosecurite.ulg.ac.be/?langue=en (accessed on 22 February 2023)) [29]. This definition seems comprehensible by trained operators/stakeholders/students/veterinarians (e.g., [22,23,24,25,26,27,28,29,30]. The simplification of this definition was translated in terms of the rule of 5 Bs [18]. However, room for improvement exists, especially in terms of better communication of the definition (e.g., appealing wording). This definition did also include environmental and public health aspects. As suggested by Renault et al. [5], in the future, the importance of biosecurity in mitigating the risks for animal and public health and environmental contamination will have to be further developed and taken into account [16,18,31].

The strengths of the survey were the inclusion of more than three hundred diverse respondents in a short period of time (one month) coming from 56 countries (five continents) with a balanced representation of both male and female respondents. Another aspect is the originality to define the agreement regarding eight existing definitions of biosecurity using a scale.

The main limitations of the survey were the absence of a sampling frame of operators, managers, and stakeholders involved in biosecurity around the world. For this reason, we opted for a snowball sampling in order to capture, as much as possible, key persons minimizing the bias. The survey highlights also the importance of diverse scientific networks (especially the ones supported by the EU-COST). In addition, the effect or the origin and category of respondents were tested to verify the possible effect of the sampling strategy. Another limitation is the over-representation of respondents with a scientific background on animal health and belonging to universities, research centers, or government officials that potentially induced a bias. Other relevant stakeholders, such as farm operators, managers, or private veterinarians, were very low in representation in the study. Indeed, the effect of the field of expertise (animal health versus other fields) and the effect of academic (university/school) staff versus other categories of respondents were tested, and no statistical difference was demonstrated for the most popular definition. However, it is desirable to extend the study on the definitions of biosecurity through more sectors and not only in the livestock sector, as well as to more categories of stakeholders. Another factor impeding the distribution of the survey to those who effectively implement biosecurity in the field, such as farmers and veterinarians, was the use of English as the main language of the survey, without alternatives in a short time. In the future, it would be ideal to translate the survey to other languages and to involve livestock farming associations in the survey dissemination. Finally, a combination of different methodologies to find a consensus on the definition of livestock biosecurity would be valuable for future surveys.

## 5. Conclusions

Participants of this survey, mainly from universities, research centers, and government institutions and with a scientific background in animal health, considered that the optimal definition of livestock biosecurity should be operational, related to animal health, and should include the keywords “prevention” and “measures”. On top of this, it would be desirable to include also aspects of public and environment health, such as including the 5 Bs, as proposed by [18].

## Figures and Tables

**Table 2:** Socio-demographic characteristics of survey respondents (N = 316).

Characteristic	Value
*Male–female gender ratio*	1.07
	Number (%)
*Age*	
18 to 24 years	12 (3.8)
25 to 39 years	112 (35.4)
40 to 54 years	136 (43.1)
55 years and over	56 (17.7)
*Professional profile*	
Member of scientific (institution) and/or academic (university/school) staff	200 (63.3)
Member of technical staff	29 (9.2)
Member of administrative staff	25 (7.9)
Student	23 (7.3)
Other	39 (12.3)
*Stakeholders’ involvement in biosecurity*	
No (corresponds to interested but not involved specifically in biosecurity)	156 (49.4)
Yes, outside the COST-Action BETTER	93 (29.4)
Yes, inside the COST-Action BETTER	67 (21.2)
*Category of stakeholders*	
Government officials (group of people with the authority to govern a country or state; a particular ministry in office; e.g., Ministry of Agriculture)	48 (30)
Workers (a person who does a specified type of work or who works in a specified way; e.g., farmer worker)	41 (25.65)
Communities (social unit with commonality such as place, norms, religion, values, customs, or identity)	12 (7.5)
Shareholders (a person who owns shares in a company)	7 (4.35)
Suppliers (person or business that provides a product or service to another entity)	7 (4.35)
Investors (entity that has an invest account)	4 (2.5)
Clients (individuals that have access to the investor account)	1 (0.65)
Other	40 (25)
Main field of expertise *	
Animal health	295 (93.35)
Human health	36 (11.4)
Environmental health	26 (8.2)
Plant health	11 (3.5)
Animal production/physiology	8 (2.5)
One Health	6 (1.9)
Food safety/food sciences	3 (0.95)
Wildlife	1 (0.3)
Other	3 (0.95)

Legend: * Several fields of expertise are possible for a same respondent. For this reason, the sum of percentages is not equal to 100.

**Table 3:** Keywords cited at least 10-fold as first, second, or third position by the respondents (in decreasing order of global occurrence).

Keyword	First	Second	Third	Total
Prevention	115	21	19	155
Measures	13	11		24
Control		16		16
Health	14			14
Spread			12	12
Bio-exclusion	11			11
Containment		11		11
Introduction		11		11
Protection		10		10
Total	153	80	31	264

**Table 4:** Characteristics of an optimal definition of livestock biosecurity presented in decreasing order (N = 316).

Characteristic	Number of Occurrences (%)
*Intrinsic quality*	
Operational	230 (72.8)
Comprehensive	179 (56.6)
Simple	176 (55.7)
Theoretical	17 (5.4)
*Aspect to be treated*	
Related to animal health	203 (64.2)
Related to public health	159 (50.3)
Related to environmental health	124 (39.2)
Related to plant health	85 (26.9)
*Others*	
Translated in a legislation	88 (27.8)

## Data Availability

The data that support the findings of this study are available from the corresponding author upon request.

## Appendix A. Initial Small Survey on the Stakeholder’s Agreement with Some Existing Definitions of Biosecurity

### Appendix A.1. Letter

Dear Participant,

Under the new COST Action “Biosecurity Enhanced Through Training Evaluation and Raising Awareness” (BETTER) CA20103, we start an initial small survey on the stakeholder’s agreement with eight existing definitions of biosecurity. These alternative definitions were extracted from various sources and publications (e.g., Renault et al., 2021; [5]).

This initial short survey will facilitate discussions on the optimal definition of livestock biosecurity in the current COST Action BETTER.

The completion of this short survey takes only few minutes.

This survey is entirely voluntary and your data will be kept anonymous.

You may also distribute this survey in your networks.

Thank you very much for your invaluable contribution.

Claude Saegerman and Jarkko Niemi

### Appendix A.2. Note on Privacy

Participation is open to persons aged 18 or older. This survey is entirely voluntary and anonymous. The data will be collected and analysed by the University of Liège, Unit of Research in Epidemiology and Risk Analysis applied to veterinary sciences with the help of other members of the above COST Action. Your data might be shared, but they will be completely anonymous, and it will not be possible to identify you individually from your answers. If you are concerned about this study or about the manner by which your data are processed, or if you wish to contact us about your rights, please first contact Claude Saegerman at the following address: claude.saegerman@uliege.be

### Appendix A.3. Questionnaire

There are 18 questions in this survey.

#### Appendix A.3.1. Part 1. Socio-Demographic Characteristics

Q1. In which country do you work or study?

Please choose only one of the following:

Afghanistan, Albania, Algeria, etc.

Other

Q2. What is your gender?

Please choose only one of the following:

Male

Female

Other

Q3. How old are you?

Please choose only one of the following:

18 to 24 years

25 to 39 years

40 to 54 years

55 years and over

Q4. Are you:

Please choose only one of the following:

A student

A member of administrative staff

A member of technical staff

A member of scientific (institution) and/or academic (university/school) staff

Other

If more than one answer is possible, please select the category you most identify with.

Q5. Are you a stakeholder in biosecurity?

Please choose only one of the following:

Yes

No

Note: A stakeholder means here an individual, group of people, members, or any organization that can be affected by the result of the action.

Q6. Are you a stakeholder of the EU-COST action BETTER?

Only answer this question if the following conditions are met:

Answer was ‘Yes’ at question ‘5 [Q4]’ (Are you a stakeholder in biosecurity?)

Please choose only one of the following:

Yes

No

Note: This action focuses on enhancing biosecurity in animal farming through training, biosecurity evaluation and awareness raising.

Q7. Which category of stakeholder are you?

Only answer this question if the following conditions are met:

Answer was ‘Yes’ at question ‘5 [Q4]’ (Are you a stakeholder in biosecurity?)

Please choose only one of the following:

Workers

Clients

Investors

Shareholders

Communities

Suppliers

Governments

Other

Note: If more than one answer is possible, please select the category you most identify with.

Q8. In which field(s) do you work?

Please choose all that apply:

Animal health

Plant health

Human health

Environmental health

Other

#### Appendix A.3.2. Part 2. Agreement with Some Definitions

Q9. Please indicate to what extent you agree with the following definition of biosecurity by using a scale from 0 (fully disagree) to 10 (fully agree). “A strategic and integrated concept that encompasses the policy and regulatory frameworks (including instruments and activities) that analyze and manage risk in food safety, public health, animal life and health, and plant life and health, including associated environmental risk”

Please choose the appropriate response for each item:

0 1 2 3 4 5 6 7 8 9 10

Q10. Please indicate, to what extent you agree with the following definition of biosecurity by using a scale from 0 (fully disagree) to 10 (fully agree). “The sum of management and physical measures designed to reduce the risk of the introduction, development and spread of diseases to, from and within: (a) an animal population, or (b) an establishment, zone, compartment, means of transport or any other facilities, premises or location”

Please choose the appropriate response for each item:

0 1 2 3 4 5 6 7 8 9 10

Q11. Please indicate, to what extent you agree with the following definition of biosecurity by using a scale from 0 (fully disagree) to 10 (fully agree). “The prevention of misuse through loss, theft, diversion or intentional release of pathogens, toxins and any other biological materials”

Please choose the appropriate response for each item:

0 1 2 3 4 5 6 7 8 9 10

Q12. Please indicate, to what extent you agree with the following definition of biosecurity by using a scale from 0 (fully disagree) to 10 (fully agree). “The vital work of strategy, efforts and planning to protect human, animal and environmental health against biological threats”

Please choose the appropriate response for each item:

0 1 2 3 4 5 6 7 8 9 10

Q13. Please indicate, to what extent you agree with the following definition of biosecurity by using a scale from 0 (fully disagree) to 10 (fully agree). “The strategies to assess and manage the risk of infectious diseases, quarantine pests, invasive alien species, living modified organisms, and biological weapons”

Please choose the appropriate response for each item:

0 1 2 3 4 5 6 7 8 9 10

Q14. Please indicate, to what extent you agree with the following definition of biosecurity by using a scale from 0 (fully disagree) to 10 (fully agree). “A unified concept to integrate human, animal, plant, and environmental health”

Please choose the appropriate response for each item:

0 1 2 3 4 5 6 7 8 9 10

Q15. Please indicate, to what extent you agree with the following definition of biosecurity by using a scale from 0 (fully disagree) to 10 (fully agree). “All measures to prevent the introduction of pathogens (bio-exclusion) and reduce the spread of pathogens (bio-containment)”

Please choose the appropriate response for each item:

0 1 2 3 4 5 6 7 8 9 10

Q16. Please indicate, to what extent you agree with the following definition of biosecurity by using a scale from 0 (fully disagree) to 10 (fully agree). “All measures: 1) to limit the risk of introduction (bio-exclusion); 2) to limit the spread of the pathogen within the same facility, e.g., by isolating excreting animals (bio-compartmentation); 3) to limit the spread of the disease agent outside the facility (inter-herd transmission) (bio-containment); 4) to prevent the risk of human contamination (bio-prevention); 5) to prevent any environmental bio-contamination and persistence of the pathogen (bio-preservation)”

Please choose the appropriate response for each item:

0 1 2 3 4 5 6 7 8 9 10

#### Appendix A.3.3. Part 3. Important Keywords to Consider for a further Consolidated Definition of Livestock Biosecurity

Q17. Please mention maximum three keywords that describe biosecurity the best (in decreasing order of importance). At least one keyword is requested.

Please write your answer(s) here:

First keyword

Second keyword

Third keywords

#### Appendix A.3.4. Part 4. Desirable Quality of an Optimal Definition of Livestock Biosecurity on Your Side

Q18. In your opinion, an optimal definition of livestock biosecurity needs to be:

Please choose all that apply:

simple

operational

theoretical

comprehensive

related to animal health

related to public health

related to plant health

related to environmental health

translated in a legislation

Thank you very much for your kind collaboration.

Claude Saegerman and Jarkko Niemi

Submit your survey.

Thank you for completing this survey.

## Appendix B. Number of Respondents by Continent and Country (Alphabetic Order)

**Continent**	**Country**	**Number of Respondents**	**Continent**	**Country**	**Number of Respondents**
Africa	Algeria	1	Europe	Bulgaria	1
	Burkina Faso	1		Croatia	3
	Cameroon	8		Denmark	1
	Chad	1		Estonia	7
	Ethiopia	2		Finland	4
	Gambia	1		France	16
	Ivory Coast	1		Germany	3
	Madagascar	1		Greece	5
	Mali	2		Ireland {Republic}	14
	Niger	13		Italy	15
	South Africa	1		Kosovo	2
America	Argentina	2		Luxembourg	1
	Brazil	1		Macedonia	4
	Canada	8		Montenegro	1
	Colombia	1		Netherlands	8
	Ecuador	22		Norway	2
	United States	9		Poland	1
Asia	Bangladesh	3		Portugal	8
	Cambodia	1		Serbia	4
	China	3		Slovakia	2
	East Timor	1		Slovenia	9
	Laos	1		Spain	21
	Thailand	4		Sweden	5
	Turkey	4		Switzerland	6
	Vietnam	1		Ukraine	1
Europe	Albania	2		United Kingdom	16
	Austria	8	Oceania	Australia	1
	Belgium	47		New Zealand	3
Legend: Two respondents were not mentioned due to international level of activities (FAO).					
